# Radiation effects on 3D rotating flow of Cu-water nanoliquid with viscous heating and prescribed heat flux using modified Buongiorno model

**DOI:** 10.1038/s41598-021-00107-x

**Published:** 2021-10-19

**Authors:** Wahib Owhaib, Mahanthesh Basavarajappa, Wael Al-Kouz

**Affiliations:** 1 Department of Mechanical and Maintenance Engineering, German Jordanian University, Amman, 11180 Jordan; 2 Center for Mathematical Needs, Department of Mathematics, CHRIST (Deemed to be University), Bangalore, Karnataka 560029 India

**Keywords:** Engineering, Mathematics and computing

## Abstract

In this article, the three-dimensional (3D) flow and heat transport of viscous dissipating Cu-H_2_O nanoliquid over an elongated plate in a rotating frame of reference is studied by considering the modified Buongiorno model. The mechanisms of haphazard motion and thermo-migration of nanoparticles along with effective nanoliquid properties are comprised in the modified Buongiorno model (MBM). The Rosseland radiative heat flux and prescribed heat flux at the boundary are accounted. The governing nonlinear problem subjected to Prandtl’s boundary layer approximation is solved numerically. The consequence of dimensionless parameters on the velocities, temperature, and nanoparticles volume fraction profiles is analyzed via graphical representations. The temperature of the base liquid is improved significantly owing to the existence of copper nanoparticles in it. The phenomenon of rotation improves the structure of the thermal boundary layer, while, the momentum layer thickness gets reduced. The thermal layer structure gets enhanced due to the Brownian movement and thermo-migration of nanoparticles. Moreover, it is shown that temperature enhances owing to the presence of thermal radiation. In addition, it is revealed that the haphazard motion of nanoparticles decays the nanoparticle volume fraction layer thickness. Also, the skin friction coefficients found to have a similar trend for larger values of rotation parameter. Furthermore, the results of the single-phase nanoliquid model are limiting the case of this study.

## Introduction

In recent years, nanoliquids have established prodigious curiosity owing to their extensive applications for example solar collectors, advanced nuclear systems, thermal storage systems, electronic chip cooling, microprocessors, hybrid-powered machines, laser-assisted drug delivery, solar liquid heating/cooling, optoelectronics, and super-efficient magnets, etc. Engineered nanoliquids are formed by mixing tiny nano-sized solid particles (Cu, Al, Au, Al_2_O_3_, TiO_2_, ZnO_2_, etc.) and base fluid (such as oil, ethylene glycol, and water). Choi and Eastman^1^ explored that suspending nanoparticles in the water had remarkably improved the thermal efficiency. Theoretical investigation of heat transport is carried out by using two well-known models, viz. Khanafer–Vafai–Lightstone (KVL) model^2^ and Buongiorno two-component nanoliquid model^3^ In the KVL model, the nanoparticles and base fluid possess the same velocity and it includes effective thermophysical properties. Whereas, the Buongiorno model (BM) considers two significant mechanisms such as Brownian motion and thermodiffusion.

Kuznetsov and Nield^4^ considered the Buongiorno two-component nanoliquid model to study the boundary layer of a nanofluid over a vertical plate with natural convection. They found that the temperature field improved due to the random movement of nanoparticles. Afterward, Nield and Kuznetsov^5^ employed the BM model to analyze the Minkowycz problem in the presence of a porous medium. They disclosed that the thermodiffusion phenomenon is favorable for thicker thermal layer thickness. Khan and Pop^6^ studied the nanoliquid dynamics over a flat elongated plate by considering the BM model and Keller box method. They established that the Brownian motion aspect reduces the nanoparticle volume fraction profile. Makinde and Aziz^7^ improvised the work of Khan and Pop^6^ by considering the convective (Newton) boundary condition. Hayat et al*.*^8^ studied the impact of Newton boundary conditions on the 3D flow of nanofluids using the BM model and couple stress fluid model. Homotopy solution for the 3D flow of nanofluid using BM model subjected to the Newton condition and magnetism is proposed by Hayat et al*.*^9^. The BM model and Oldroyd-B fluid model are utilized by Hayat et al*.*^10^ to study the 3D stretched flow of nanofluid subjected to magnetism. For a more detailed review of the 3D flow of nanofluid using BM model studies reader can refer to references^11–15^.

The studies in Refs.^4–15^ are more or less similar to the heat and mass transfer problem with the thermodiffusion aspect. Also, the effectual thermophysical properties of nanoliquid affect the flow distributions significantly, hence they can’t be ignored and are better to include to obtain accurate results. In this direction, Yang et al*.*^16^ used the Buongiorno nanoliquid model along with effectual thermo-physical properties of nanoliquids to study the convective thermal transport of nanofluids. Later, this model is well known as the Modified Buongiorno Model (MBM). Malvandi et al*.*^17^ used the MBM to examine the completely developed flow of nanofluids in an annular pipe. They concluded that the single-phase nanoliquid model results can be recovered from MBM. Malvandi and Ganji^18^ examined the dynamics of nanofluids in a microchannel by using the MBM model. However, the studies related to nanofluid flow using Modified Buongiorno Model are very limited.

The significance of thermal radiation in many industrial and engineering processes like electric power, non-destructive testing, solar cell panels, and many others is vital. Therefore, it is very crucial to comprehend the aspect of thermal radiation to attain the desired quality of products in industrial processes. Sheikholeslami and Rokni^19^ studied the importance of Rosseland thermal radiation and Coulomb force on the dynamics of nanofluids in an enclosure saturated by porous space. They found that the thermal radiation process supports improving the thermal layer thickness. Mahmoud and Megahed^20^ studied the significance of thermal radiation on the dynamics of non-Newtonian fluid with mixed convection and thermal diffusion. The impact of Rosseland radiative heat is examined by Das et al*.*^21^ on the dynamics of nanoliquid in a micro-channel. Shehzad et al*.*^22^ investigated to study the thermal radiative heat transfer and 3D flow of nano Jeffrey fluid with magnetism.

Recently, Raza et al*.*^23^ and Wakif et al.^24^ are investigated the significance and applications of the thermal radiation aspect. Moreover, Alam et al*.*^25^ used the turbulent SST model to capture the heat transfer characteristics of micro-pin-fin. Several researchers numerically solved the derived 3D flow and energy equations of nanofluids or hybrid nanofluid by the Runge–Kutta–Fehlberg-method (RKF). Furthermore, Baslem et al*.*^26^ investigated the thermal behavior of porous fin fully wetted with various nanofluids under natural convection and radiation condition. They found that Cu-water was best to enhance the fin heat transfer amongst the investigated nanofluids Al_2_O_3_-water and TiO_2_-water. In addition. Ganesh Kumar et al.^27^ explained heat transfer behavior influenced by both tangent hyperbolic nanofluid and magnetic field over a moving stretched surface. Additionally, Punith Gowda et al.^28^ presented a 3D nonlinear model for nanofluid flow over an expansion and contraction of a rotating disk featured with thermophoretic particle deposition. In addition, Ahmadian et al*.*^29^ used homotopy solution and PCM to show the influence of 3D unsteady flow caused by the wavy rotating disk and magnetic field on hybrid nanofluids Ag/MgO-Water. Ahmadian et al.^30^ studied the Brownian motion and thermophoresis effect on maxwell nanofluid between two stretchable horizontal rotating disks under a magnetic field. They solved their model by boundary value solver (Bvp4c) and RK4. Finally, Lv et al*.*^31^ used PCM to explore the impact of thermal radiation, magnetic field, and the upshot of Hall current on flow and heat transfer characteristics of carbon nanotubes, and iron ferrite nanofluids flow over a spinning disk.

Inspired by the above-indicated literature and applications, the prime purpose of the current research is to study the 3D rotating flow of nanoliquid over a stretched plate using the modified Buongiorno model (MBM). The modified Buongiorno model comprised haphazard movement and thermo-migration of nanoparticles along with effectual thermophysical properties. The influences of viscous heating, thermal radiation, and prescribed surface heat flux boundary conditions are also scrutinized. The nonlinear partial differential boundary value problem is solved numerically and the results are analyzed. Further, the velocity, temperature, heat transport rate, and mass transport rate are examined for various parameters.

## Mathematical formulation

**Figure 1:**
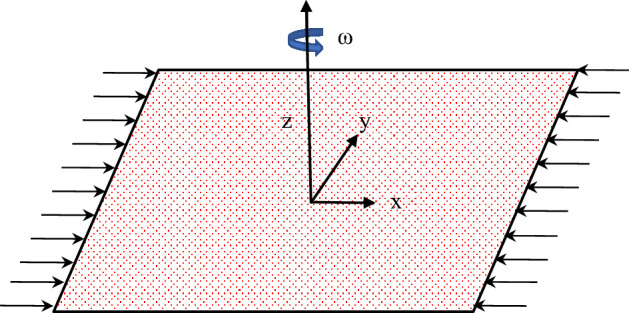
Schematic diagram of the problem under consideration.

The rotating 3D of water-based *Cu* nanoliquid over a stretched plate subjected to a rotating frame is considered. The surface heat flux boundary conditions are included. Figure 1 represents a schematic diagram for the problem under investigation.

The transport mechanism for nanoliquid has been addressed by the Modified Buongiorno Model (MBM) that includes the effective nanoliquid properties, haphazard motion of $Cu$ nanoparticles, and thermo-migration mechanism. The rectangular coordinate framework is aligned with $xy$−plane and the fluid region is considered at $z \ge 0$. The $Cu$ nanoliquid rotates unvaryingly about $z$-axis with an unvarying rate $\omega$. To characterize the effective thermal conductivity and effectual dynamic viscosity of nanoliquid, the Maxwell-Garnetts^2^ and Brinkman^3^ models are utilized. These models correspond to spherical shape nanoparticles of the volume fraction less than 5–6%. Utilizing the above-mentioned assumptions, and following Lund et al*.*^32^, the subsequent boundary-layer expressions are (see^3,6,9,16^);

1 $$\frac{\partial u}{\partial x}+\frac{\partial v}{\partial y}+\frac{\partial w}{\partial z}=0,$$

2 $${\rho }_{nl}\left(u\frac{\partial u}{\partial x}+v\frac{\partial u}{\partial y}+w\frac{\partial u}{\partial z}-2\omega v\right)={\mu }_{nl}\frac{{\partial }^{2}u}{\partial {z}^{2}},$$

3 $${\rho }_{nl}\left(u\frac{\partial v}{\partial x}+v\frac{\partial v}{\partial y}+w\frac{\partial v}{\partial z}+2\omega u\right)={\mu }_{nl}\frac{{\partial }^{2}v}{\partial {z}^{2}},$$

4 $${\left(\rho {C}_{p}\right)}_{nl}\left(u\frac{\partial T}{\partial x}+v\frac{\partial T}{\partial y}+w\frac{\partial T}{\partial z}\right)={k}_{nl}\frac{{\partial }^{2}T}{\partial {z}^{2}}+{\left(\rho {C}_{p}\right)}_{np}\left\{{D}_{B}\frac{\partial T}{\partial z}\frac{\partial C}{\partial z}+\frac{{D}_{T}}{{T}_{\infty }} {\left(\frac{\partial T}{\partial z}\right)}^{2}\right\}+{\mu }_{nl}\left({\left(\frac{\partial u}{\partial z}\right)}^{2}+{\left(\frac{\partial v}{\partial z}\right)}^{2}\right)-\frac{\partial {q}_{R}}{\partial z}$$

5 $$u\frac{\partial C}{\partial x}+v\frac{\partial C}{\partial y}+w\frac{\partial C}{\partial z}={D}_{B}\frac{{\partial }^{2}C}{\partial {z}^{2}}+\frac{{D}_{T}}{{T}_{\infty }}\frac{{\partial }^{2}T}{\partial {z}^{2}}.$$

The last term from the right-hand side of Eq. (4) corresponds to the thermal radiation term and it is modeled by using Rosseland’s approximation. The ${q}_{R}$ (radiative heat flux) is given below (see^11^)

6 $${q}_{R}=-\frac{4\alpha }{3\beta }\frac{\partial {T}^{4}}{\partial z}.$$

Upon simplifying Eq. (6) through low temperature difference yields (see^11^)

7 $$\frac{\partial {q}_{R}}{\partial z}=-\frac{16\alpha {T}_{\infty }^{3}}{3\beta }\frac{{\partial }^{2}T}{\partial {z}^{2}}.$$

Using Eq. (7) in Eq. (4)

8 $${\left(\rho {C}_{p}\right)}_{nl}\left(u\frac{\partial T}{\partial x}+v\frac{\partial T}{\partial y}+w\frac{\partial T}{\partial z}\right)={k}_{nl}\frac{{\partial }^{2}T}{\partial {z}^{2}}+{\left(\rho {C}_{p}\right)}_{np}\left\{{D}_{B}\frac{\partial T}{\partial z}\frac{\partial C}{\partial z}+\frac{{D}_{T}}{{T}_{\infty }} {\left(\frac{\partial T}{\partial z}\right)}^{2}\right\}+{\mu }_{nl}\left({\left(\frac{\partial u}{\partial z}\right)}^{2}+{\left(\frac{\partial v}{\partial z}\right)}^{2}\right)+\frac{16\alpha {T}_{\infty }^{3}}{3\beta }\frac{{\partial }^{2}T}{\partial {z}^{2}},$$

where $u, v$ and $w$ are velocities along $x, y$ and $z$‐directions, $\nu =\frac{\mu }{\rho }$ is the kinematic viscosity, $\mu$ is the dynamic viscosity, $\rho$ is the density, $T$ is the temperature, $C$ is the dimensionless nanoparticle volume fraction, $\alpha =\frac{k}{\rho {C}_{p}}$ is the thermal diffusivity, $k$ is the thermal conductivity, $\rho {C}_{p}$ is the specific heat, ${D}_{B}$ is the coefficient of Brownian diffusion, ${D}_{T}$ is the coefficient of thermo-migration diffusion, $\alpha$ is the Stefan–Boltzmann constant and $\beta$ is the mean absorption factor. The boundary conditions are

9 $$\left.\begin{array}{l}w=v=0, {u}_{w}=u(x)=ax, -{k}_{nl}\left(\frac{\partial T}{\partial z}\right)={q}_{w}, C={C}_{w} at z=0, \\ \\ v=0, u=0, T={T}_{\infty }, C={C}_{\infty } as z\to \infty \end{array}\right\},$$

where $a>0$ is stretching rate and ${q}_{w}=\left({T}_{w}-{T}_{\infty }\right){k}_{l}\sqrt{a/{\nu }_{l}}$ is constant heat flux.

The effective density and specific heat of water-based $Cu$ nanoliquid are as follows:

$${\rho }_{nl}=\left(1-\phi \right){\rho }_{l}+\phi {\rho }_{np} ,$$

10 $${\left({\rho C}_{p}\right)}_{nl}=\left(1-\phi \right)(\rho {{C}_{p})}_{l}+\phi (\rho {{C}_{p})}_{np },$$

Brinkman model for dynamic viscosity and Maxwell model for thermal conductivity are used.

11 $$\frac{{\mu }_{nl}}{{\mu }_{l}}=\frac{1}{{\left(1-\phi \right)}^{2.5}},$$

12 $$\frac{{k}_{nl}}{{k}_{l}}=\frac{{k}_{np}+2{k}_{l}-2\phi \left({k}_{l}-{k}_{np}\right)}{{k}_{np}+2{k}_{l}+\phi \left({k}_{l}-{k}_{np}\right)},$$

The subscripts $l, nl,$ and $np$ indicate base liquid, nanoliquid, and nanoparticles correspondingly. Because of Eqs. (10)–(12) and considering

$$\zeta =z\sqrt{\frac{{{u}_{w}}}{{x\nu }_{l}}}, u=ax{f}^{{\prime}}\left(\zeta \right), v=axg\left(\zeta \right), w=-\sqrt{{\nu }_{l}a}f\left(\zeta \right),$$

13 $$T=\left({T}_{w}-{T}_{\infty }\right)\theta \left(\zeta \right)+{T}_{\infty }, C=\left({C}_{w}-{C}_{\infty }\right)\Theta \left(\zeta \right)+{C}_{\infty }.$$

Equation (1) is satisfied and Eqs. (2), (3), (5), (8) and (9) are reduced to

14 $$\frac{{\Psi }_{2}}{{\Psi }_{1}}{f}^{{{\prime}}{{\prime\prime}}}+f{f}^{{^{\prime}}{^{\prime}}}-{{f}^{{\prime}}}^{2}+2Rog=0,$$

15 $$\frac{{\Psi }_{2}}{{\Psi }_{1}}{{g}^{{{\prime\prime}}}}+f{g}^{{\prime}}-gf{^{\prime}}-2Rof{^{\prime}}=0,$$

16 $$\frac{{\Psi }_{4}+Rd}{Pr}{\theta }^{{{\prime\prime}}}+{\Psi }_{3}f{\theta }^{{\prime}}+Nb{\Theta }^{{\prime}}{\theta }^{{\prime}}+Nt{{\theta }^{{\prime}}}^{2}+{\Psi }_{2}Ec \left[{\left({f}^{{^{\prime}}{^{\prime}}}\right)}^{2}+{\left(g{^{\prime}}\right)}^{2} \right]=0,$$

17 $${\Theta }^{{^{\prime}}{^{\prime}}}+\frac{Nt}{Nb}{\theta }^{{{\prime\prime}}}+LePrf{\Theta }^{{\prime}}=0,$$

18 $$\left.\begin{array}{c}f=0, g=0, {f}^{{\prime}}=1, {\theta }^{{\prime}}=\frac{-1}{{\Psi }_{4}}, \Theta =1 at \zeta =0 \\ \\ {f}^{{\prime}}=0, g=0, \theta =0, \Theta =0 as \zeta \to \infty . \end{array} \right\},$$

where $\zeta$ -similarity variable, $f, g,$ $\theta ,$ and $\Theta$ are dimensionless axial velocity, transverse velocity, temperature, and nanoparticle volume fraction correspondingly, prime denotes the differentiation with respect to $\zeta$, $\mathit{Pr}=\frac{{\left({C}_{p}\mu \right)}_{l}}{{k}_{l}}$ (Prandtl number), $Ro=\frac{\omega }{a}$ (rotation parameter), $Le=\frac{{\alpha }_{l}}{{D}_{B}}$ (Lewis number), $Nt=\frac{{\left(\rho {C}_{p}\right)}_{np}{D}_{T}\left({T}_{w}-{T}_{\infty }\right)}{{\left(\rho {C}_{p}\right)}_{l}{T}_{\infty }{\nu }_{l}}$ (thermophoresis parameter), $Nb=\frac{{\left(\rho {C}_{p}\right)}_{np}{D}_{B}\left({C}_{w}-{C}_{\infty }\right) }{{\left(\rho {C}_{p}\right)}_{l}{\nu }_{l}}$ (Brownian motion parameter), $Rd=\frac{16\alpha {T}_{\infty }^{3}}{3{k}_{l}\beta }$ (thermal radiation parameter), $Ec=\frac{{u}_{w}^{2}}{{{C}_{p}}_{l}\left({T}_{w}-{T}_{\infty }\right)}$ (Eckert number),

$${\Psi }_{1}=\left(1-\phi \right)+\phi \frac{{\rho }_{np}}{{\rho }_{l}},$$

$${\Psi }_{2}=\frac{1}{{\left(1-\phi \right)}^{2.5}},$$

$${\Psi }_{3}=\left(1-\phi \right)+\phi (\rho {{C}_{p})}_{np }/(\rho {{C}_{p})}_{l}, \mathrm{and}$$

$${\Psi }_{4}=({k}_{np}+2{k}_{l}-2\phi ({k}_{l}-{k}_{np}))/({k}_{np}+2{k}_{l}+\phi ({k}_{l}-{k}_{np})).$$

The expressions of dimensionless local friction factors (${Sf}_{x} \& {Sf}_{y}$), local Nusselt number $(N{u}_{x})$ and local Sherwood number $(S{h}_{x})$ are

$$R{e}_{x}^{0.5}{Sf}_{x}={\Psi }_{2}f{^{\prime}}{^{\prime}}\left(0\right),$$

$$R{e}_{x}^{0.5}{Sf}_{y}={\Psi }_{2}g{^{\prime}}\left(0\right),$$

$$R{e}_{x}^{-0.5}N{u}_{x}=-\frac{{\Psi }_{4}\left(1+Rd\right)}{\theta \left(0\right)},$$

19 $$R{e}_{x}^{-0.5}S{h}_{x}=-{\Theta }^{{\prime}}\left(0\right),$$

where $R{e}_{x}=\frac{{{x}}u_w}{{\nu }_{l}}$ is local Reynolds number.

## Numerical technique

The defined nonlinear problem in Eqs. (14)–(18) is solved numerically using the Finite Difference Method based bvp5c alogorithm. Now set $f={y}_{1}, {f}^{{\prime}}={y}_{2}, {f}^{{^{\prime}}{^{\prime}}}={y}_{3}, g={y}_{4}, {g}^{{\prime}}={y}_{5}, \theta ={y}_{6}$, ${\theta }^{{\prime}}={y}_{7}$, $\Theta ={y}_{8}$ and ${\Theta }^{{\prime}}={y}_{9}$ to obtain the following single-order differential system:

20 $${y}_{1}^{{\prime}}={y}_{2},$$

21 $${y}_{2}^{{\prime}}={y}_{3},$$

22 $${y}_{3}^{{\prime}}=\frac{{\Psi }_{2}}{{\Psi }_{1}}\left({y}_{2}^{2}-{y}_{1}{y}_{3}-2Ro{y}_{4}\right),$$

23 $${y}_{4}^{{\prime}}={y}_{5},$$

24 $${y}_{5}^{{\prime}}=\frac{{\Psi }_{2}}{{\Psi }_{1}}\left(2Ro{y}_{2}+{y}_{2}{y}_{4}-{y}_{1}{y}_{5}\right),$$

25 $${y}_{6}^{{\prime}}={y}_{7},$$

26 $${y}_{7}^{{\prime}}=\frac{-\mathrm{Pr}\left\{{\Psi }_{3}{y}_{1}{y}_{7}+Nb{y}_{7}{y}_{9}+Nt{\left({y}_{7}\right)}^{2}+{\Psi }_{2}Ec({y}_{3}^{2}+{y}_{5}^{2})\right\}}{{\Psi }_{4}+Rd},$$

27 $${y}_{8}^{{\prime}}={y}_{9},$$

28 $${y}_{9}^{{\prime}}=-LePr{{y}_{1}y}_{10}+\left(\frac{Nt}{Nb}\right)\left(\frac{\mathrm{Pr}\left\{{\Psi }_{3}{y}_{1}{y}_{7}+Nb{y}_{7}{y}_{9}+Nt{\left({y}_{7}\right)}^{2}+{\Psi }_{2}Ec\left({y}_{3}^{2}+{y}_{5}^{2}\right)\right\}}{{\Psi }_{4}+Rd}\right),$$

with

$${y}_{1}\left(0\right)=0,{y}_{4}\left(0\right)=0, {y}_{2}\left(0\right)=1, {y}_{8}\left(0\right)=1, {y}_{7}\left(0\right)=\frac{-1}{{\Psi }_{4}} ,$$

29 $${y}_{2}\left(\infty \right)=0, {y}_{4}\left(\infty \right)=0, {y}_{6}\left(\infty \right)=0, {y}_{7}\left(\infty \right)=0.$$

The above system is solved using the bvp5c routine of MATLAB (see Shampine et al*.* ^33^). This routine integrates a system of differential equations of the form y′ = f(x,y) specified, subject to the boundary conditions. Details of the algorithm used in the solution are provided in the “Appendix” section of this article. This method is used extensively by many researchers to solve the nonlinear problem, further, they have validated the method used.

## Interpretation of the outcomes

**Figure 2:**
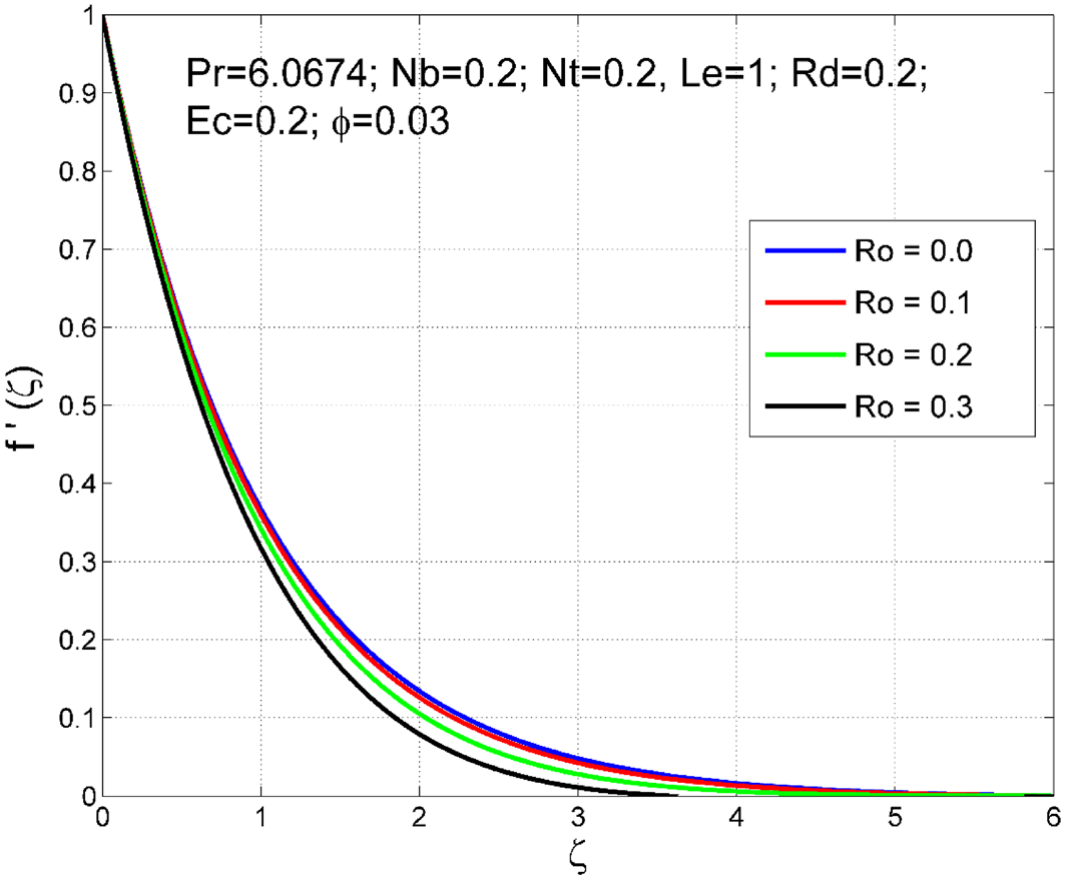
Effect of $Ro$ on $f{^{\prime}}(\zeta )$.

**Figure 3:**
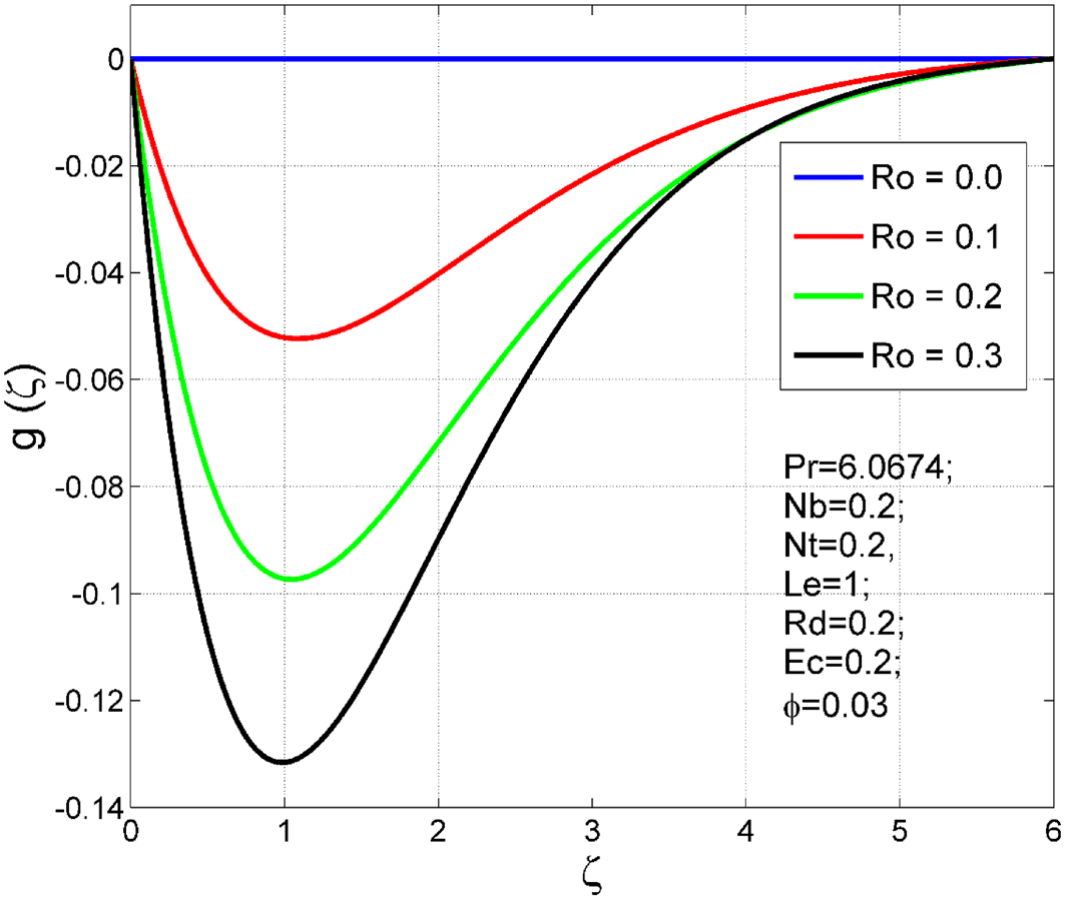
Effect of $Ro$ on $g(\zeta )$.

**Figure 4:**
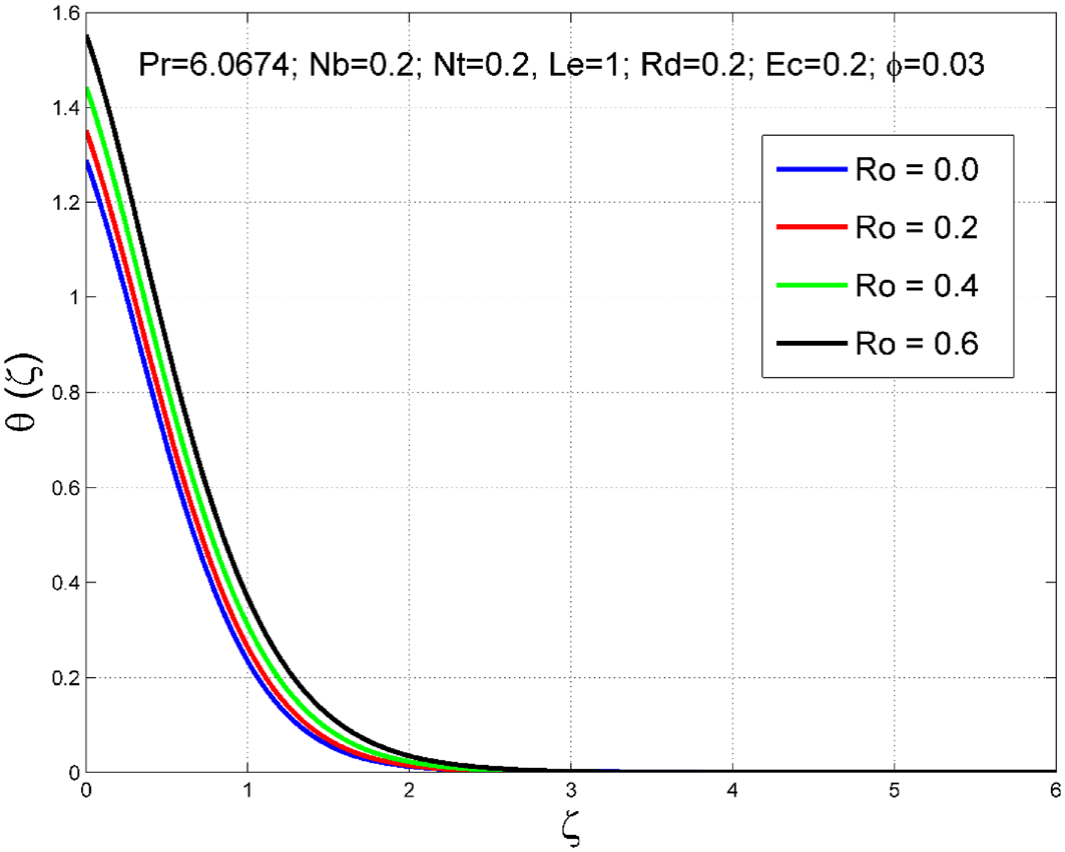
Effect of $Ro$ on $\theta (\zeta )$.

**Figure 5:**
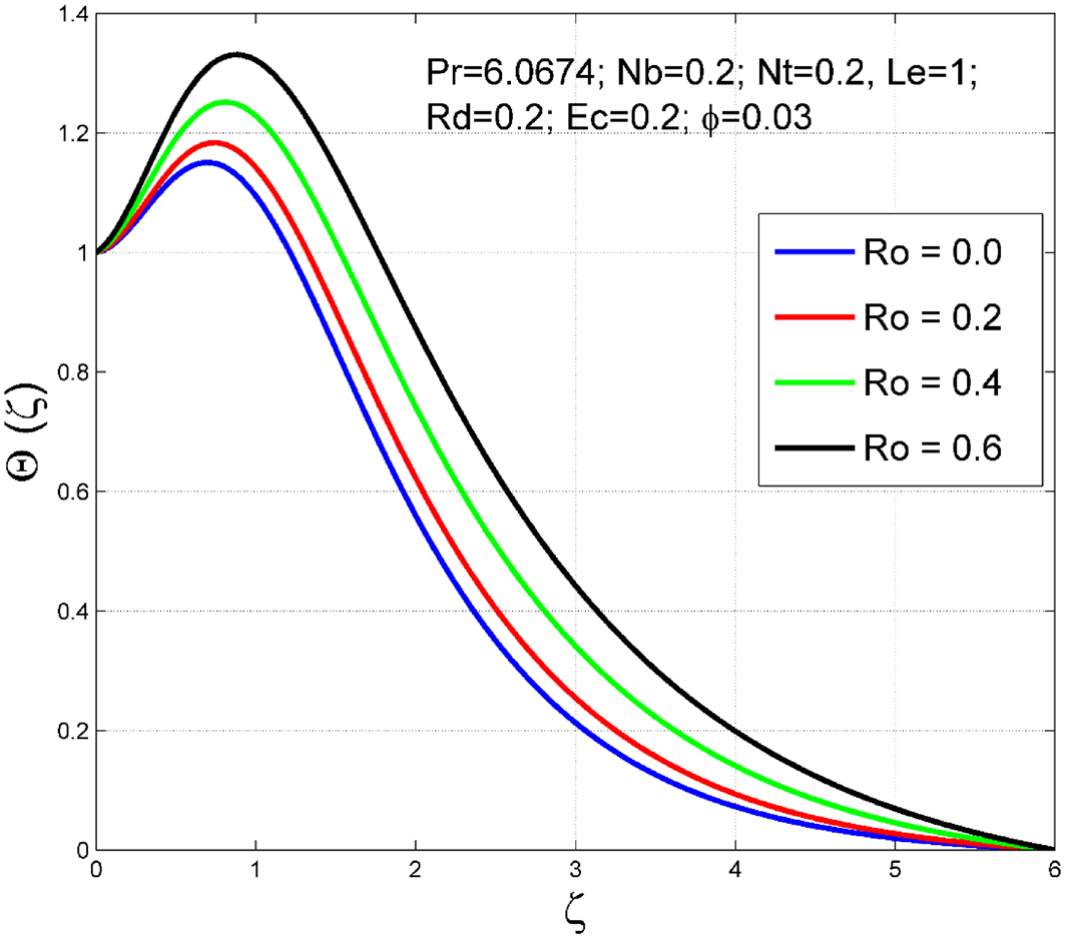
Effect of $Ro$ on $\Theta (\zeta )$.

**Figure 6:**
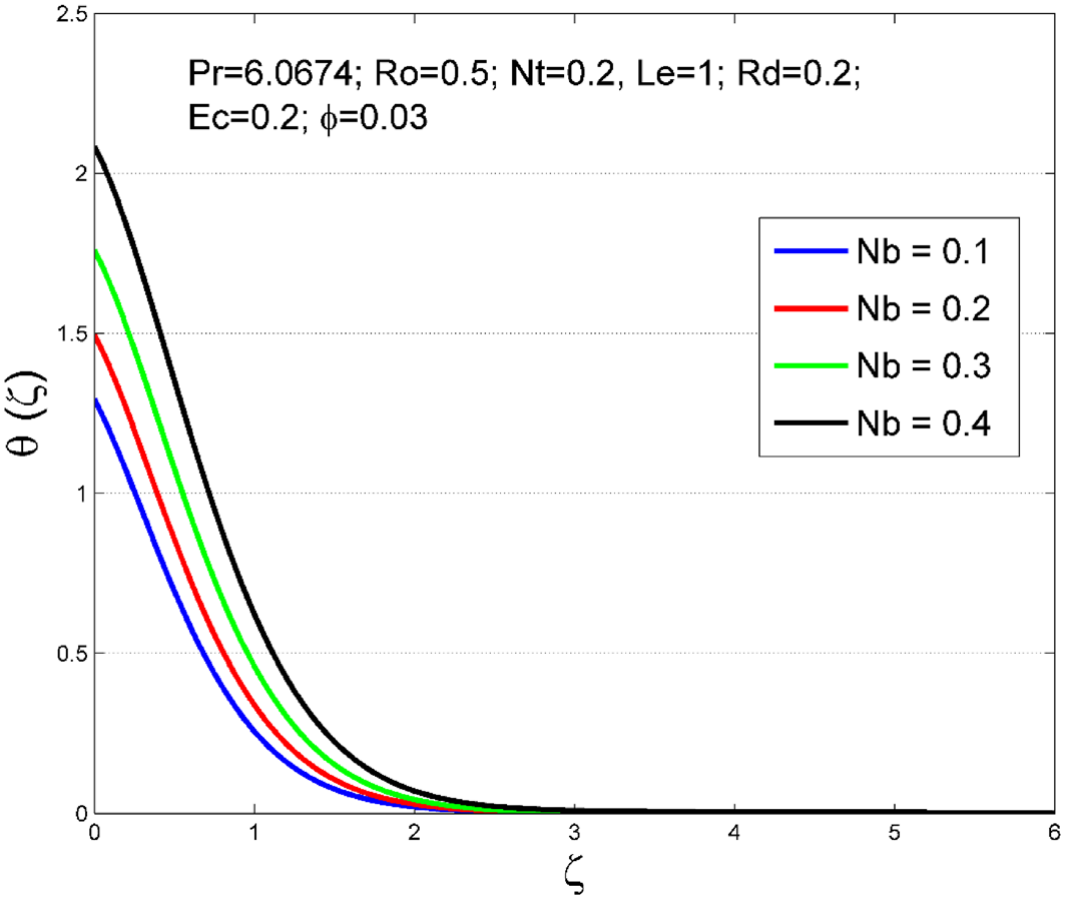
Effect of $Nb$ on $\uptheta (\zeta )$.

**Figure 7:**
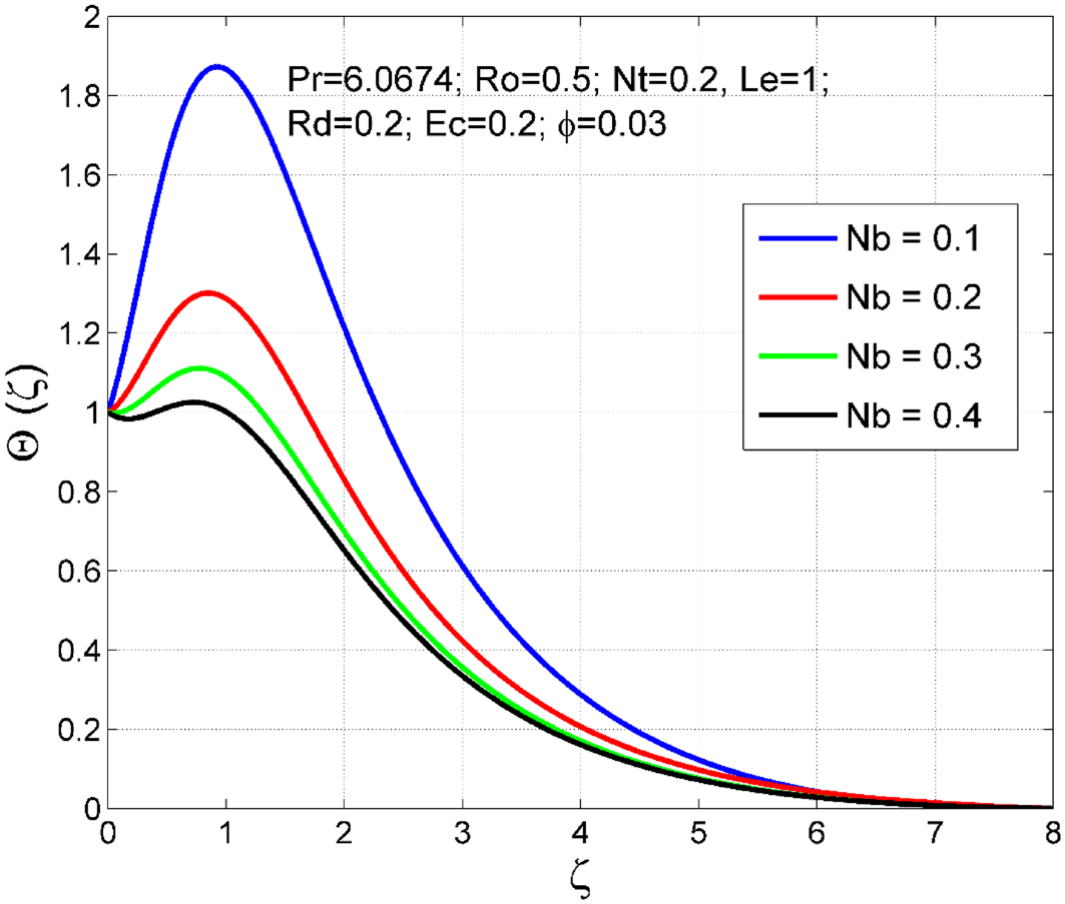
Effect of $Nb$ on $\Theta (\zeta )$.

**Figure 8:**
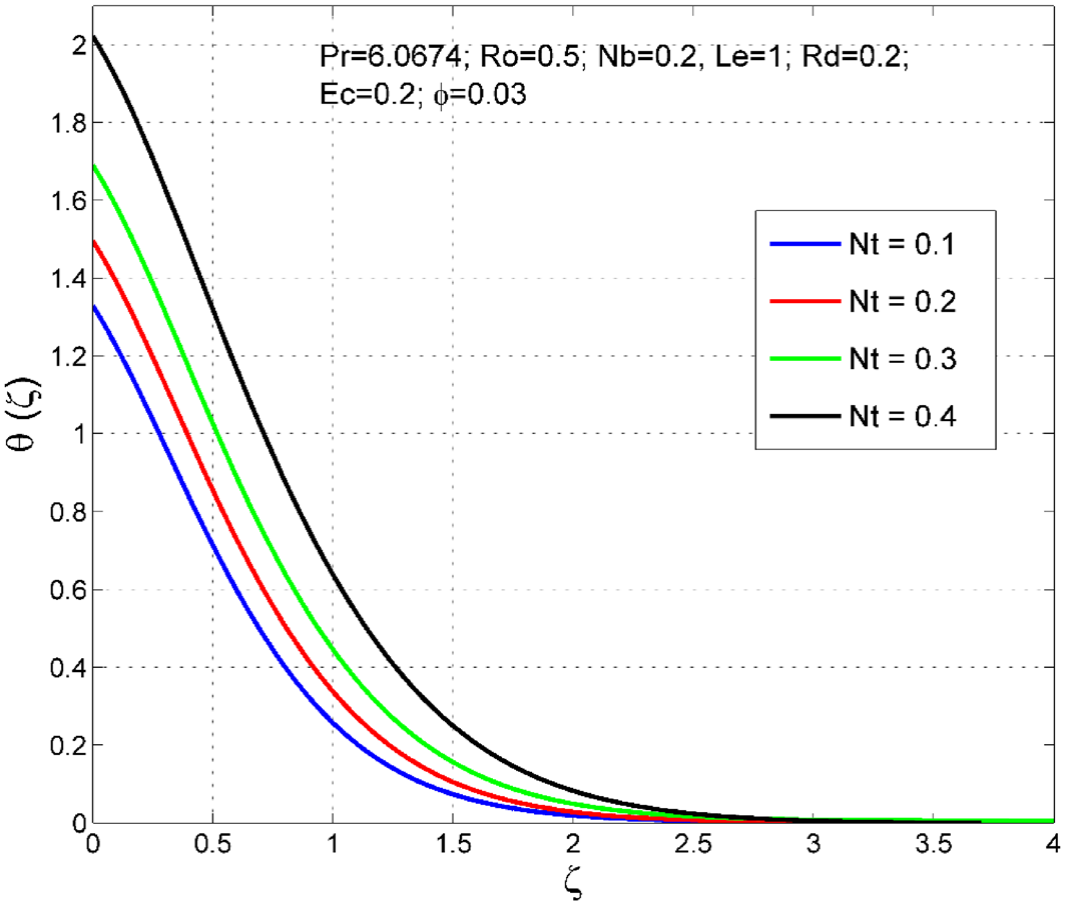
Effect of $Nt$ on $\uptheta (\zeta )$.

**Figure 9:**
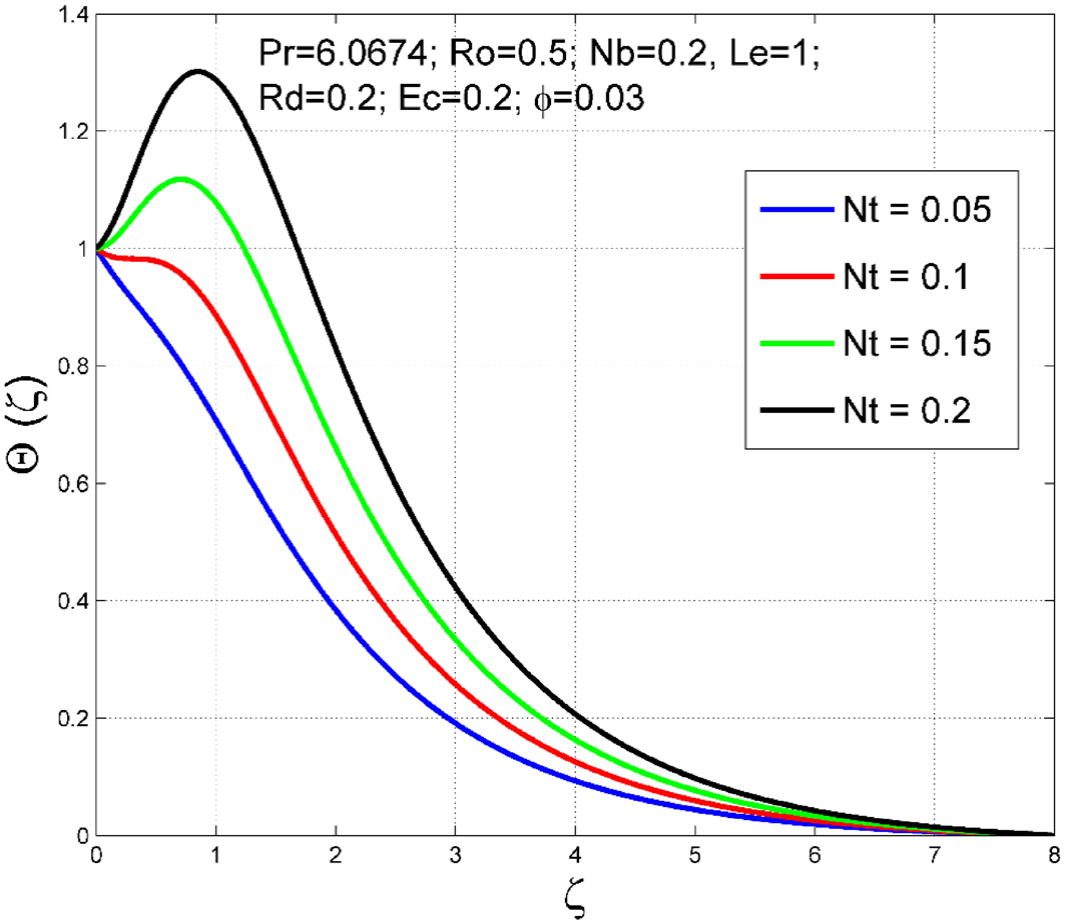
Effect of $Nt$ on $\Theta (\zeta )$.

**Figure 10:**
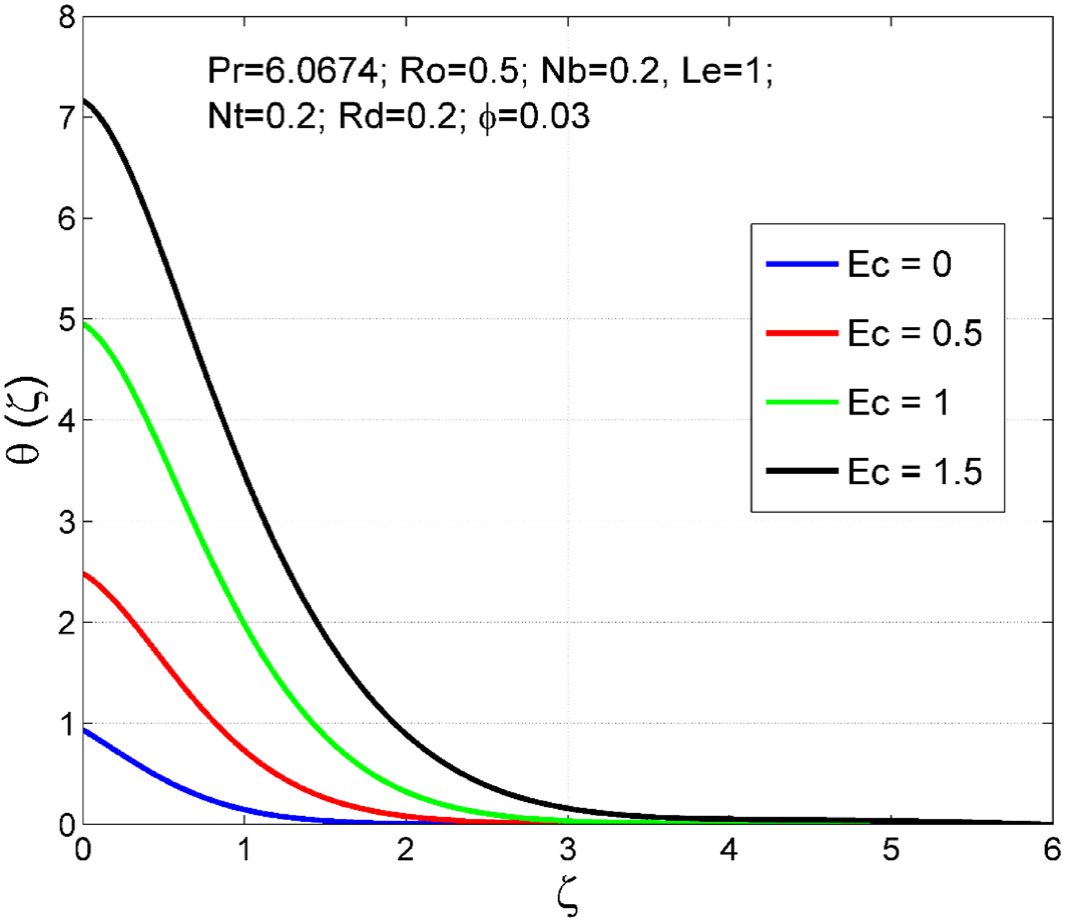
Effect of $Ec$ on $\uptheta (\zeta )$.

**Figure 11:**
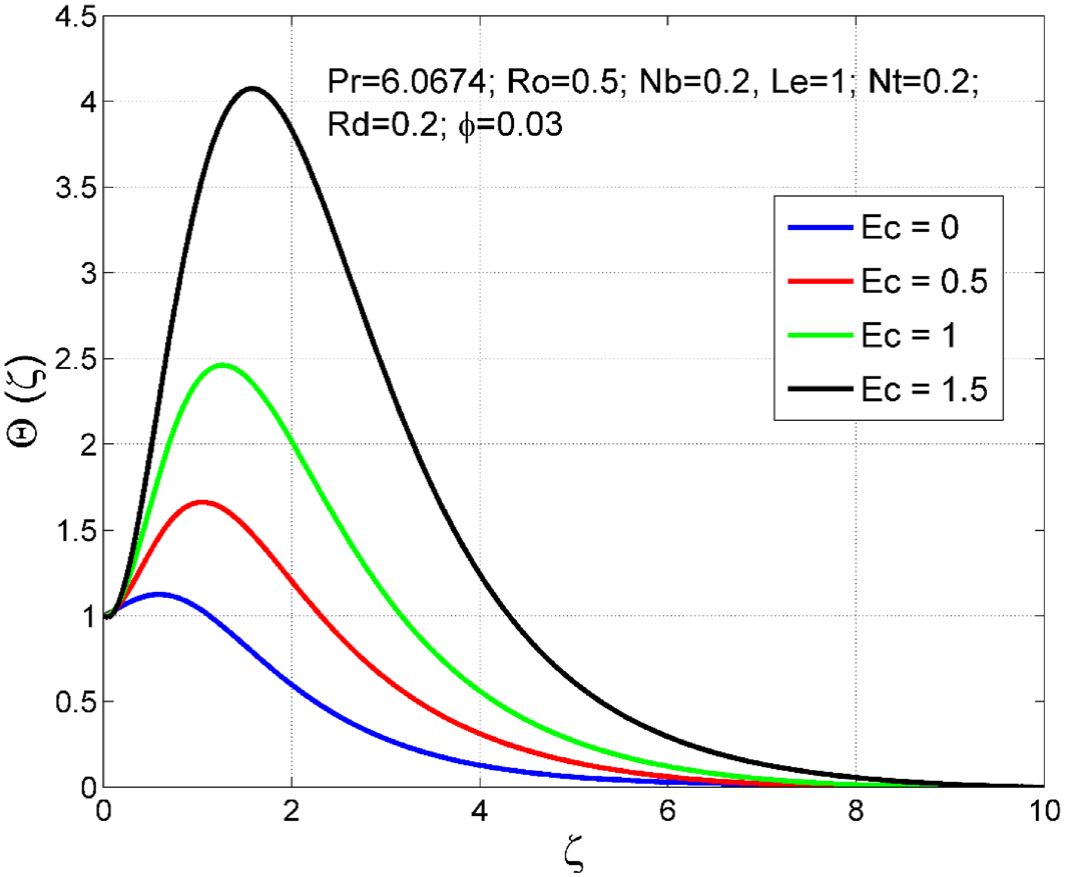
Effect of $Ec$ on $\Theta (\zeta )$.

**Figure 12:**
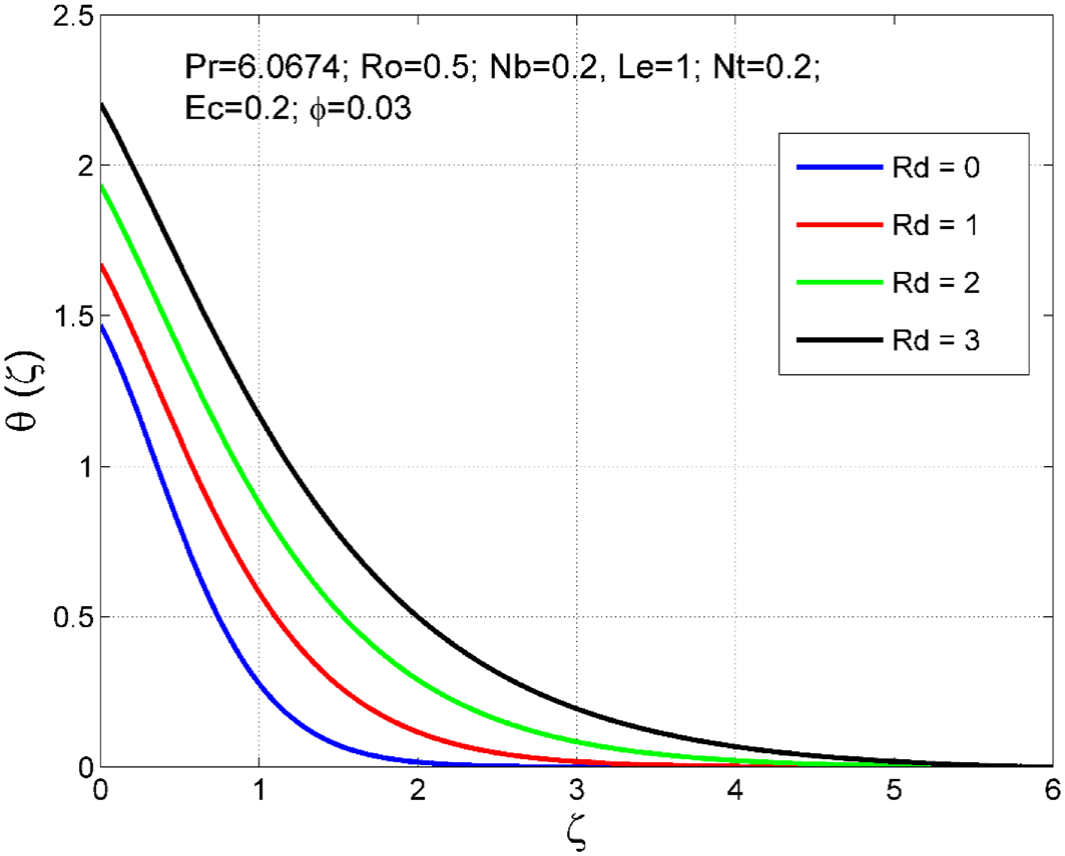
Effect of $Rd$ on $\uptheta (\zeta )$.

**Figure 13:**
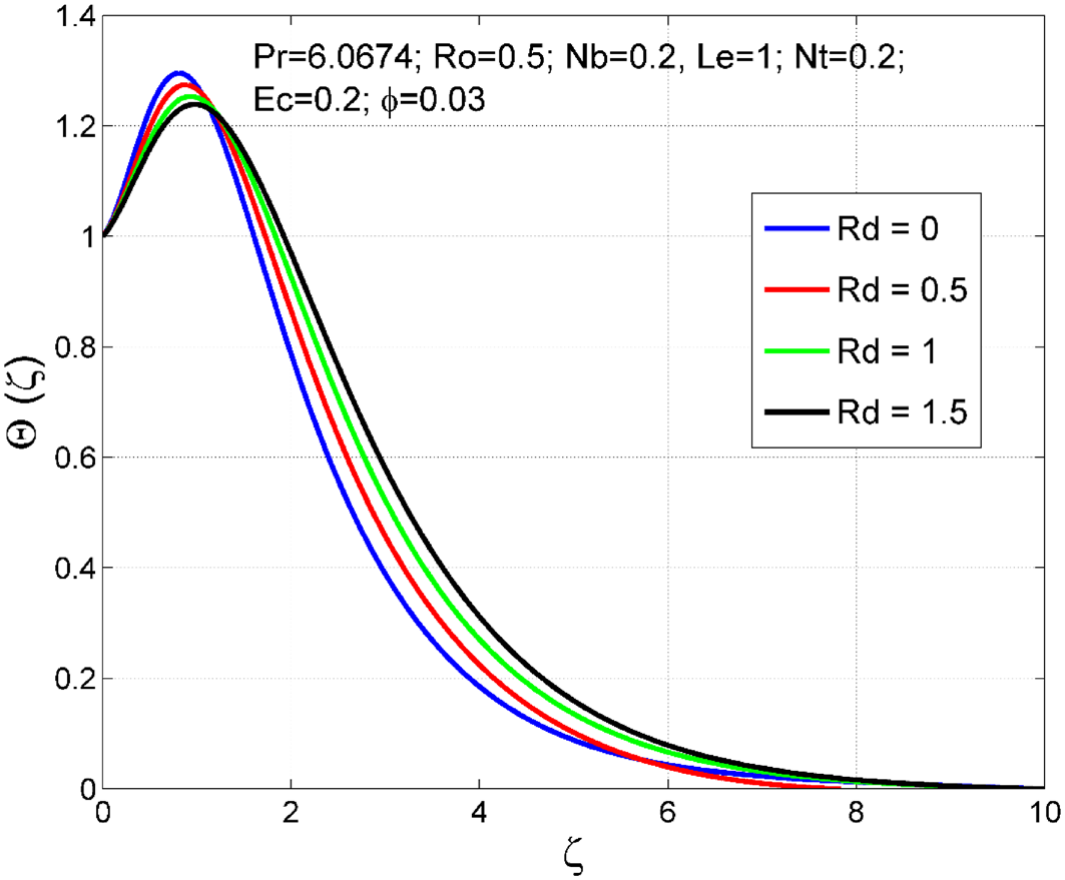
Effect of $Rd$ on $\Theta (\zeta )$.

**Figure 14:**
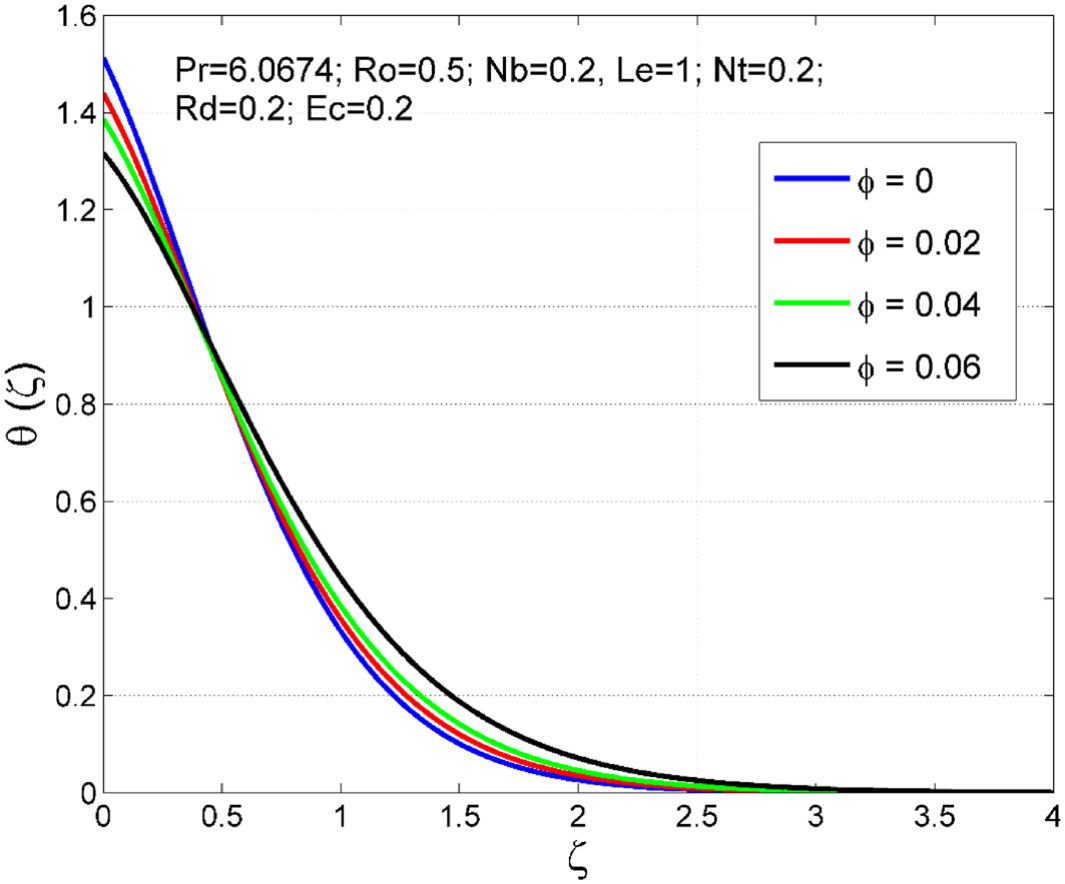
Effect of $\phi$ on $\uptheta (\zeta )$.

**Figure 15:**
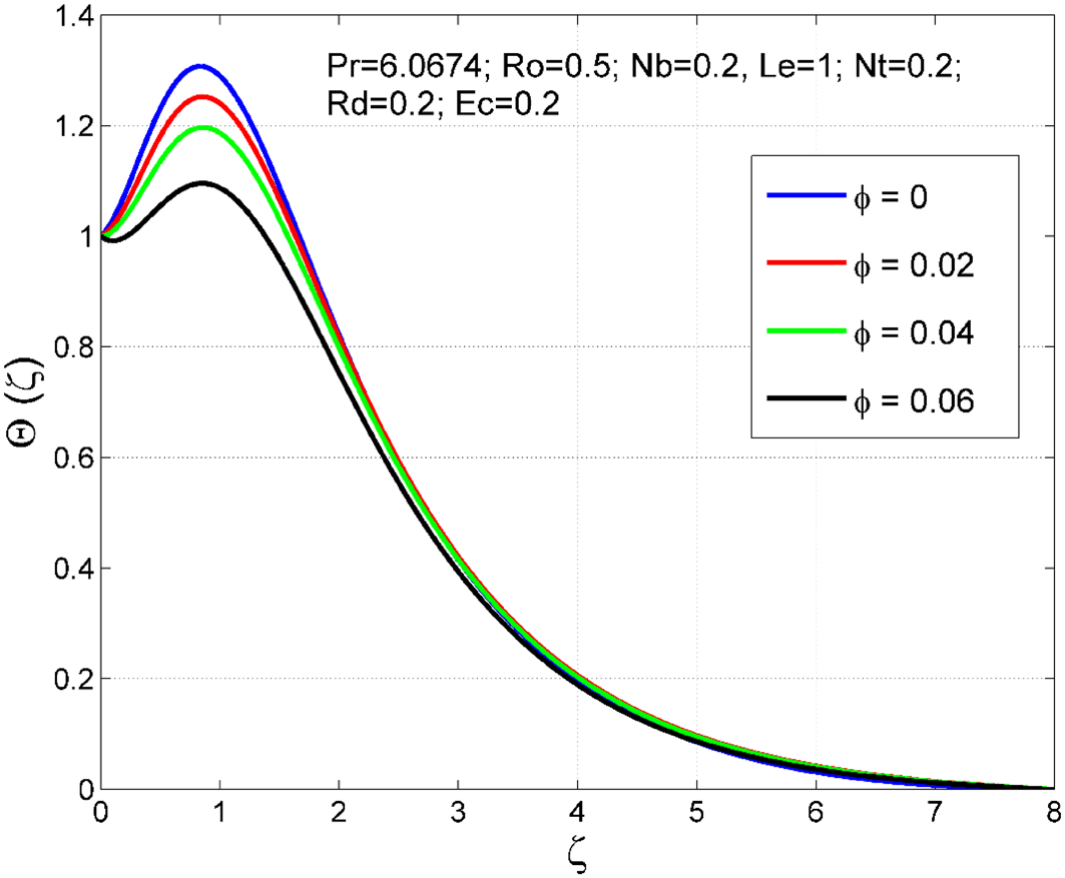
Effect of $\phi$ on $\Theta (\zeta )$.

**Figure 16:**
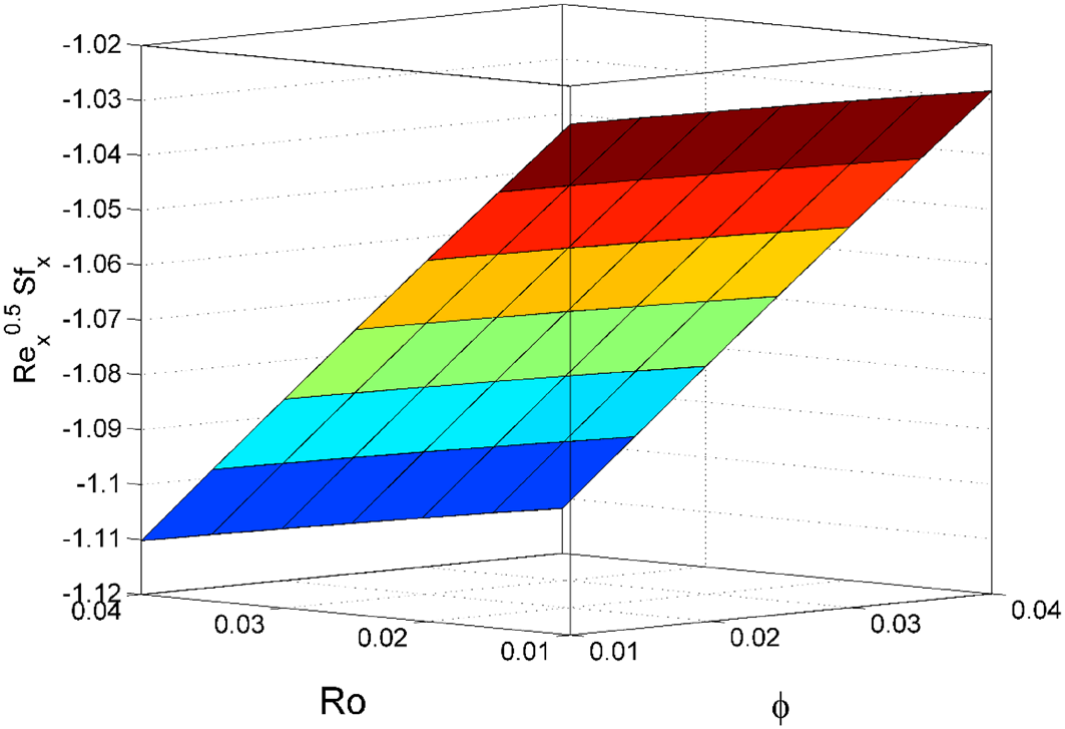
Effect of $\phi$ and $Ro$ on ${\mathrm{Re}}_{\mathrm{x}}^{0.5}S{f}_{x}$.

**Figure 17:**
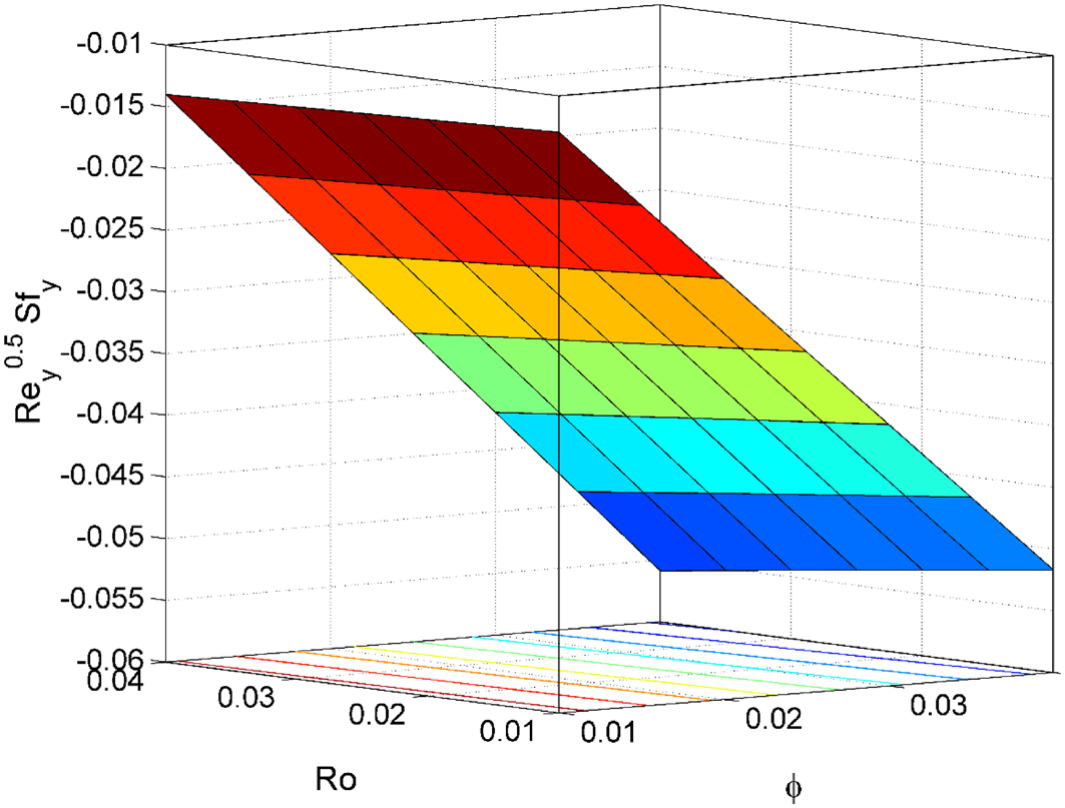
Effect of $\phi$ and $Ro$ on ${\mathrm{Re}}_{\mathrm{y}}^{0.5}S{f}_{y}$.

**Figure 18:**
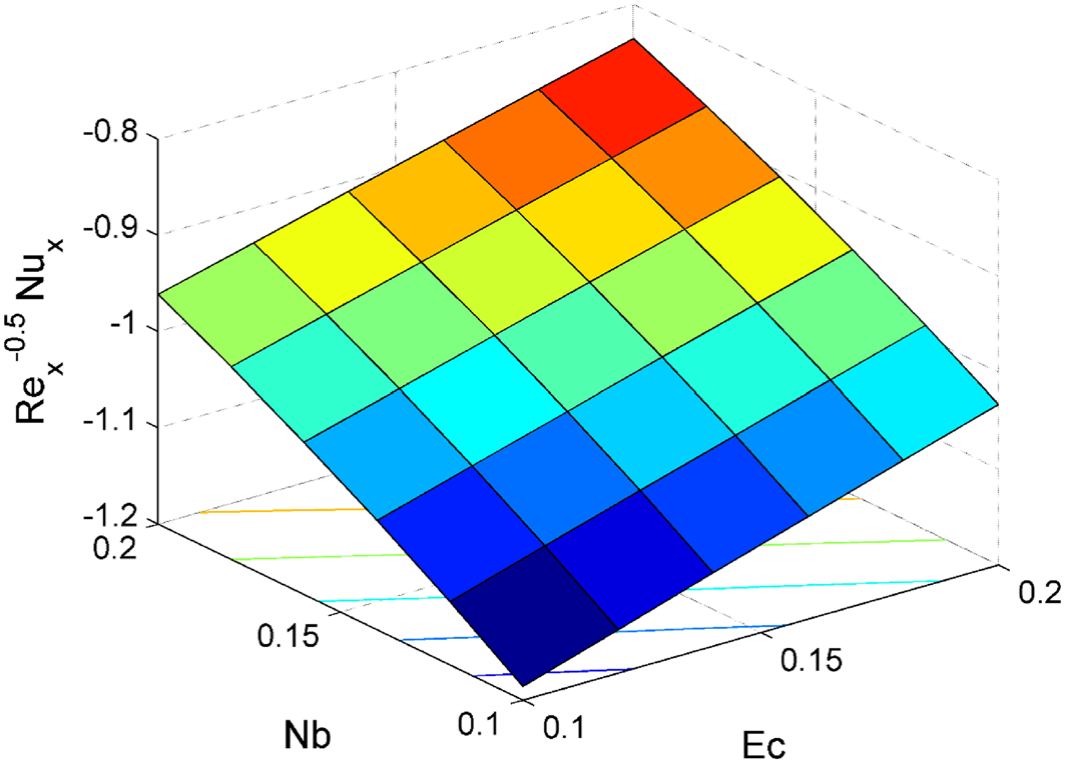
Effect of $Nb$ and $Ec$ on ${\mathrm{Re}}_{\mathrm{x}}^{-0.5}N{u}_{x}$.

**Figure 19:**
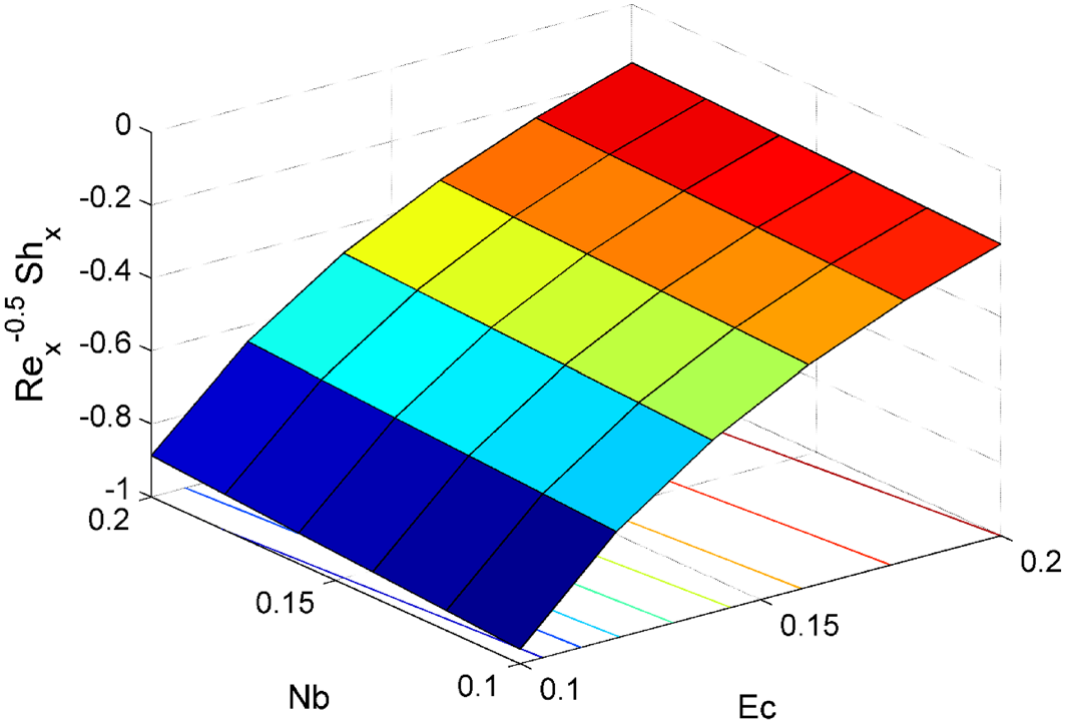
Effect of $Nb$ and $Ec$ on ${\mathrm{Re}}_{\mathrm{x}}^{-0.5}S{h}_{x}$.

**Figure 20:**
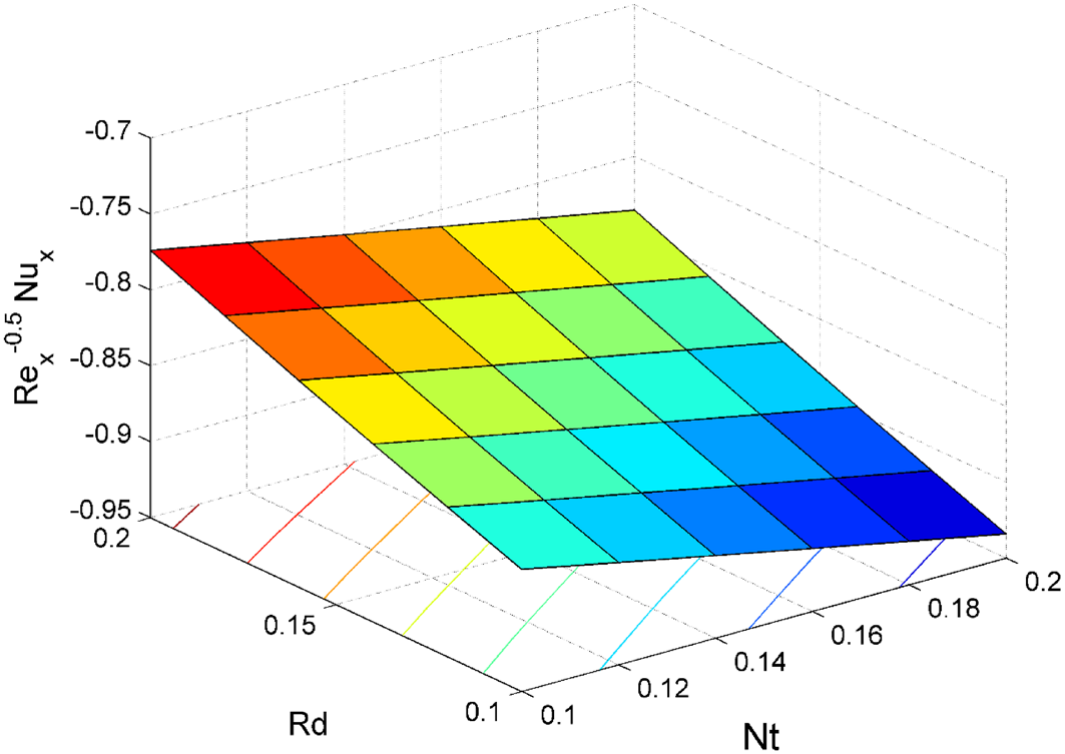
Effect of $Rd$ and $Nt$ on ${\mathrm{Re}}_{\mathrm{x}}^{-0.5}N{u}_{x}$.

**Figure 21:**
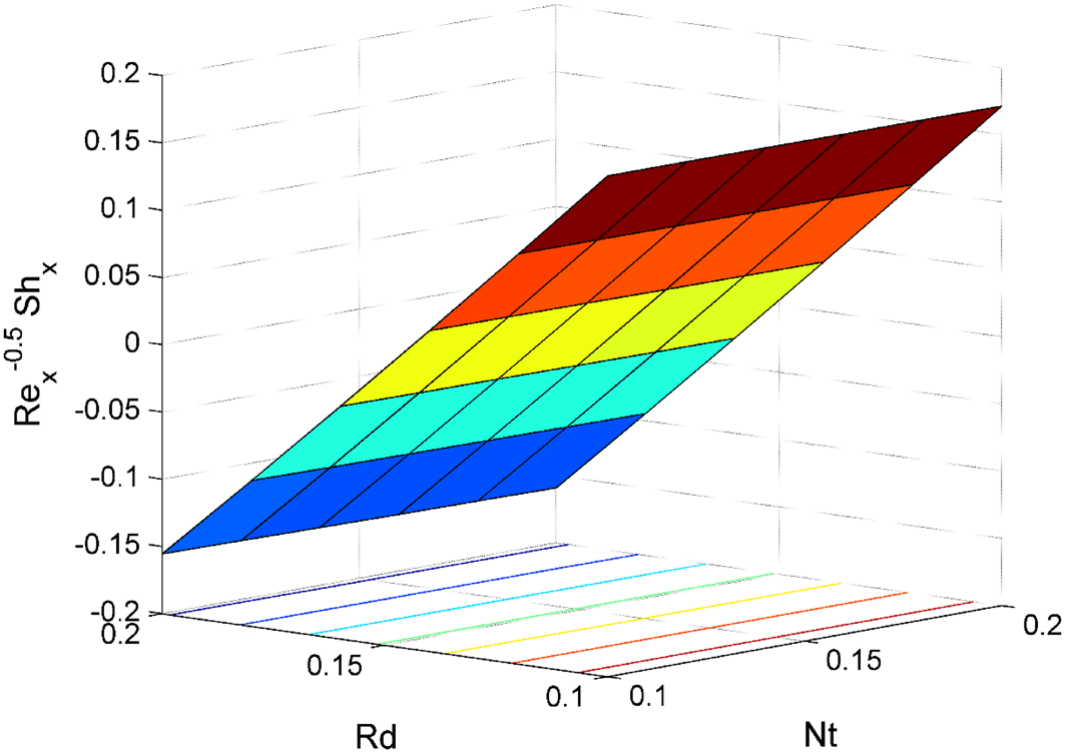
Effect of $Rd$ and $Nt$ on ${\mathrm{Re}}_{\mathrm{y}}^{-0.5}S{h}_{x}$.

Figures 2, 3, 4, 5, 6, 7, 8, 9, 10, 11, 12, 13, 14, 15, 16, 17, 18, 19, 20 and 21 are presented to analyze the significance of numerous physical parameters such as rotation parameter ($Ro$), Brownian motion parameter ($Nb$), thermo-migration parameter ($Nt$), Eckert number ($Ec$), Rosseland radiation parameter ($Rd$), and copper nanoparticles volume fraction ($\phi$) on velocities $({f}^{{\prime}}\left(\zeta \right), g(\zeta ))$, temperature $(\theta (\zeta ))$, nanoparticle volume fraction $(\Theta (\zeta ))$, friction coefficients at the plate $(R{e}_{x}^{0.5}S{f}_{x} \& R{e}_{x}^{0.5}S{f}_{y})$, Sherwood number ($R{e}_{x}^{-0.5}S{h}_{x}$) and Nusselt number ($R{e}_{x}^{-0.5}N{u}_{x}$) distributions. The numerical simulations are carried out for $Pr=6.0674, Ro=0.5, Nb=Nt=0.2, Le=1, Rd=0.2, Ec=0.2$, and $\phi =3\%$.

The stimulus of rotation factor ($Ro$) on velocities $({f}^{{\prime}}\left(\zeta \right), g(\zeta ))$, temperature $(\theta (\zeta ))$, and nanoparticle volume fraction $(\Theta (\zeta ))$ is presented in Figs. 2, 3, 4 and 5 respectively. The magnitude of axial velocity ${f}^{{\prime}}\left(\zeta \right)$ and transverse velocity decreases by increasing values of rotation factor ($Ro$). The rotation factor ($Ro$) is a ratio of angular velocity to stretching rate. Larger values of $Ro$ indicate to lower stretching rate, due to which, the velocities are diminished for larger $Ro$. Further, in the absence of rotation ($Ro=0$) there is no $g(\zeta )$ and the ${f}^{{\prime}}\left(\zeta \right)$ is found to be higher. An increasing trend is observed for the thermal and nanoparticle volume fraction layer structure when $Ro$ values are increased (see Figs. 4, 5). Figures 6 and 7 describe how the haphazard movement of nanoparticles ($Nb$) affects the temperature $(\theta (\zeta ))$ and nanoparticle volume fraction $(\Theta (\zeta ))$ fields. A diminishing trend of nanoparticle volume fraction $(\Theta (\zeta ))$ is perceived for advanced values of Nb, while, an opposite trend is observed for temperature $(\theta (\zeta ))$ profile. The haphazard collision of suspended nanoparticles in water produces supplementary heat in the system and thereby the magnitude of the temperature field increases.

The consequence of $Nt$ on temperature $(\theta (\zeta ))$ and nanoparticle volume fraction $(\Theta (\zeta ))$ distributions are delineated in Figs. 8 and 9. The thermodiffusion factor is a quantity that measures the intensity of force produced by the thermal gradient in the liquid system. As $Nt$ increases, the additional internal heat supplied to the liquid system via thermal gradient increases, and thereby both the temperature $(\theta (\zeta ))$ and nanoparticle volume fraction $(\Theta (\zeta ))$ are enhanced.

Figures 10 and 11 display the impact of viscous heating ($Ec$) on temperature $(\theta (\zeta ))$ and nanoparticle volume fraction $(\Theta (\zeta ))$. A significant improvement occurred in both $\theta (\zeta )$ and $\Theta \left(\zeta \right)$ via higher $Ec$. Higher $Ec$ corresponds to stronger kinetic energy which causes an enhancement in the magnitude of temperature and volume fraction of $Cu$ nanoparticles. Figures 12 and 13 are drawn to visualize the influence of Rosseland thermal radiation ($Rd$) on temperature $(\theta (\zeta ))$ and nanoparticle volume fraction $(\Theta (\zeta ))$. Both $\theta (\zeta )$ and $\Theta \left(\zeta \right)$ are upsurged for increasing numeric values of $Rd$. Physically, the factor of mean absorption has an inverse relation with $Rd$. Consequently, larger $Rd$ implies a lower mean absorption factor which improves supplementary heat, and as a result, nanoliquid temperature is enhanced. Figures 14 and 15 are drawn to examine the influence of $\phi$ on $\theta (\zeta )$ and $\Theta \left(\zeta \right)$. Enhancing tendency of temperature $\theta (\zeta )$ and nanoparticle volume fraction $\Theta \left(\zeta \right)$ is seen for larger values of $\phi$. The thermal conductivity of base liquid water enhances by conveying $Cu$ nanoparticles. Stronger thermal diffusivity is responsible for the development of thermal and solute layer structures.

Figures 16 and 17 are plotted to analyze the behavior of friction factors at the plate along $$x$$ and $$y$$ directions ($$R{e}_{x}^{0.5}S{f}_{x}, R{e}_{x}^{0.5}S{f}_{y})$$, Nusselt number $${\mathrm{Re}}_{\mathrm{x}}^{-0.5}N{u}_{x}$$ and Sherwood number $$R{e}_{x}^{-0.5}S{h}_{x}$$ when $$Nb=Nt=0.2, Le=1, Pr= 6.0674, Ro=0.5, Ec=0.2, \phi =3\%,$$ and $$Rd=0.2$$ except when they are diverse. Figures 16 and 17 depict the effects of $$Ro$$ and $$\phi $$ on $$R{e}_{x}^{0.5}S{f}_{x}$$ and $$R{e}_{x}^{0.5}S{f}_{y}$$. Figure 16 exhibit that $$R{e}_{x}^{0.5}S{f}_{x}$$ decreases for larger $$Ro$$ and $$\phi $$. A similar trend is observed for $$R{e}_{x}^{0.5}S{f}_{y}$$ when $$Ro$$ and $$\phi $$ are gets increased. Figure 18 designate that the $${\mathrm{Re}}_{\mathrm{x}}^{-0.5}N{u}_{x}$$ is an increasing property of both $$Nb$$ and $$Ec$$. Figure 19 demonstrates the impact of $$Nb$$ and $$Ec$$ on $$R{e}_{x}^{-0.5}S{h}_{x}$$. It is seen that $$R{e}_{x}^{-0.5}S{h}_{x}$$ enhances for larger values of $$Nb$$ and $$Ec$$. Figures 20 and 21 explain the behavior of the Nusselt number $${\mathrm{Re}}_{\mathrm{x}}^{-0.5}N{u}_{x}$$ and Sherwood number $$R{e}_{x}^{-0.5}S{h}_{x}$$ for distinct values of $$Nt$$ and $$Rd$$. We have seen that $${\mathrm{Re}}_{\mathrm{x}}^{-0.5}N{u}_{x}$$ enhances for larger $$Rd$$ while it shows the decaying trend for greater values of $$Nt$$. Further, the $$R{e}_{x}^{-0.5}S{h}_{x}$$ reduces for increasing values of $$Rd$$.

## Final remarks

The key outcomes of the present study are:

- The temperature is found to be higher due to constant heat flux condition.
- The viscous dissipation effect leads to an enhancement of nanoparticle volume fraction and temperature profiles.
- The rotation of the plate exhibits decreasing behavior of velocity components.
- The nanoparticle volume fraction revelations increasing behavior of thermal and solute layer thickness.
- Temperature enhances owing to the presence of thermal radiation.
- The temperature profile and its related boundary layer thickness improve for larger Brownian movement parameter and thermophoresis parameter.
- The haphazard motion of nanoparticles decays the nanoparticle volume fraction layer thickness.
- Skin friction coefficients have a similar trend for larger values of rotation parameter.

## Figures and Tables

**Chart 1:**
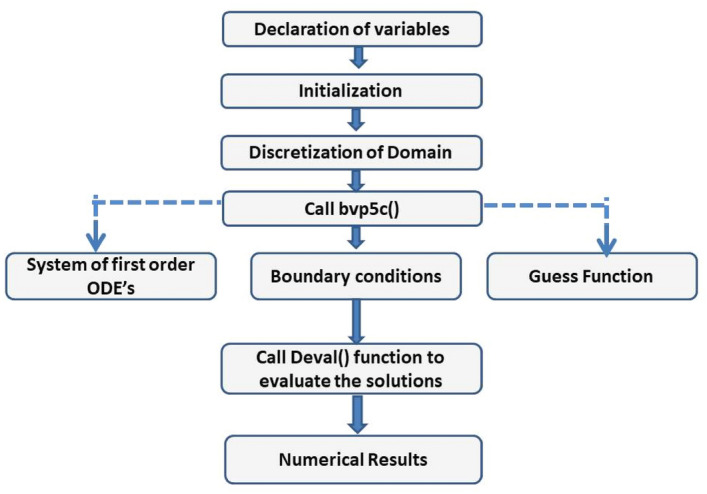
Algorithm of bvp5c method to solve boundary value problem.

## Appendix

The system of linear ordinary differential equations is solved using bvp5c function using MATLAB software. It is essential to convert the nonlinear differential equations into the first-order system of equation. Then, the resultant first-order differential equations are treated using bvp5c() function. The basic syntax of bvp5c function is $sol=bvp4c(@OdeBVP, @OdeBC, solinit, options)$ where the four important arguments are defined as:

⦁ $@OdeBVP$ function that evaluates the differential equations $f(x,y)$ and can be written in the form of $dydx=OdeBVP(x,y,parameters)$.

⦁ $@OdeBC$ A function that computes the residual in the boundary conditions $bc(y\left(a\right),y(b))$. It can be written as $res=OdeBC(ya,yb,parameters)$.

⦁ $solinit$ The guess structure formed with the helper function $bvpinit$ and can be written in the form of $solinit=bvpinit(linspace\left(etaMin, etaMax, stepsize\right), @OdeInit1)$.

⦁ $options$ Optional integration argument. A structure which create using the bvpset function. It can have the form $options=bvpset({^{\prime}}name{1}^{{\prime}}, value1,{^{\prime}}name{2}^{{\prime}}, value1,\dots$).

The algorithm of the bvp5c method is described in the Chart 1.
