# Author Correction: Radiation effects on 3D rotating flow of Cu‑water nanoliquid with viscous heating and prescribed heat flux using modified Buongiorno model

**DOI:** 10.1038/s41598-021-01794-2

**Published:** 2021-11-11

**Authors:** Wahib Owhaib, Mahanthesh Basavarajappa, Wael Al-Kouz

**Affiliations:** 1 Department of Mechanical and Maintenance Engineering, German Jordanian University, Amman, 11180 Jordan; 2 Center for Mathematical Needs, Department of Mathematics, CHRIST (Deemed to be University), Bangalore, Karnataka 560029 India

Correction to: *Scientific Reports* 10.1038/s41598-021-00107-x, published online 19 October 2021

The original version of this Article contained errors.

Firstly, the original version of this Article contained an error in Affiliation 2, which was incorrectly given as ‘Department of Mathematics, Center for Mathematical Needs, CHRIST (Deemed to be University), Bangalore, Karnataka 560029, India.’

The correct affiliation is listed below:

Center for Mathematical Needs, Department of Mathematics, CHRIST (Deemed to be University), Bangalore, Karnataka 560029, India.

Additionally, the original version of this Article contained errors in the equations.

In the Mathematical formulation section,

9 $$\left. {\begin{array}{*{20}l} {w = v = 0, u = u\left( x \right) = ax, - k_{nl} \left( {\frac{\partial T}{{\partial z}}} \right) = q_{w} , C = C_{w}\,at\,z = 0,} \hfill \\ {v = 0, u = 0, T = T_{\infty } , C = C_{\infty }\, as\, z \to \infty } \hfill \\ \end{array} } \right\}$$

now reads:

9 $$\left. {\begin{array}{*{20}l} {w = v = 0, u_{w} = u\left( x \right) = ax, - k_{nl} \left( {\frac{\partial T}{{\partial z}}} \right) = q_{w} , C = C_{w}\, at\, z = 0,} \hfill \\ {v = 0, u = 0, T = T_{\infty } , C = C_{\infty }\, as\, z \to \infty } \hfill \\ \end{array} } \right\}$$

13 $$\begin{aligned} \zeta & = z\sqrt {\frac{u}{{x\nu_{l} }}} , u = axf^{{\prime }} \left( \zeta \right), v = axg\left( \zeta \right), w = - \sqrt {\nu_{l} a} f\left( \zeta \right) \\ T & = \left( {T_{w} - T_{\infty } } \right)\theta \left( \zeta \right) + T_{\infty } , C = \left( {C_{w} - C_{\infty } } \right){\Theta }\left( \zeta \right) + C_{\infty } \\ \end{aligned}$$

now reads:

13 $$\begin{aligned} \zeta & = z\sqrt {\frac{{u_{w} }}{{x\nu_{l} }}} , u = axf^{{\prime }} \left( \zeta \right), v = axg\left( \zeta \right), w = - \sqrt {\nu_{l} a} f\left( \zeta \right) \\ T & = \left( {T_{w} - T_{\infty } } \right)\theta \left( \zeta \right) + T_{\infty } , C = \left( {C_{w} - C_{\infty } } \right){\Theta }\left( \zeta \right) + C_{\infty } \\ \end{aligned}$$

16 $$\frac{{{\Psi }_{4} + Rd}}{Pr}\theta^{{\prime \prime }} + {\Psi }_{3} f\theta^{{\prime }} + Nb{\Theta }^{{\prime }} \theta^{{\prime }} + Nt\theta^{{{\prime 2}}} + {\Psi }_{2} Ec\left( {\left( {f^{{\prime \prime }} } \right)^{2} + \left( {g^{{\prime }} } \right)^{2} } \right) = 0,$$

now reads:

16 $$\frac{{{\Psi }_{4} + Rd}}{Pr}\theta^{{\prime \prime }} + {\Psi }_{3} f\theta^{{\prime }} + Nb{\Theta }^{{\prime }} \theta^{{\prime }} + Nt\theta^{{{\prime 2}}} + {\Psi }_{2} Ec\left[ {\left( {f^{{\prime \prime }} } \right)^{2} + \left( {g^{{\prime }} } \right)^{2} } \right] = 0,$$

18 $$\left. {\begin{array}{*{20}l} {f = 0, g = 0, f^{{\prime }} = 1, \theta^{{\prime }} = \frac{ - 1}{{{\Psi }_{4} }}, \Theta = 1\, at\, \zeta = 0} \hfill \\ {f^{{\prime }} = 0, f = 0, \theta = 0, \Theta = 0\, as\, \zeta \to \infty .} \hfill \\ \end{array} } \right\}$$

now reads:

18 $$\left. {\begin{array}{*{20}l} {f = 0, g = 0, f^{{\prime }} = 1, \theta^{{\prime }} = \frac{ - 1}{{{\Psi }_{4} }}, \Theta = 1\, at\, \zeta = 0} \hfill \\ {f^{{\prime }} = 0, g = 0, \theta = 0, \Theta = 0\, as\, \zeta \to \infty .} \hfill \\ \end{array} } \right\}$$

“The expressions of dimensionless local friction factors ($Sf_{x} \& Sf_{y}$), local Nusselt number $\left( {Nu_{x} } \right)$ and local Sherwood number $\left( {Sh_{x} } \right)$ are

19 $$\begin{aligned} &Re_{x}^{0.5} Sf_{x} = {\Psi }_{2} f^{{{\prime \prime }}} \left( 0 \right), \hfill \\ &Re_{x}^{0.5} Sf_{y} = {\Psi }_{2} g^{{\prime }} \left( 0 \right), \hfill \\ &Re_{x}^{ - 0.5} Nu_{x} = - \frac{{{\Psi }_{4} \left( {1 + Rd} \right)}}{\theta \left( 0 \right)}, \hfill \\ &Re_{x}^{ - 0.5} Sh_{x} = - {{\Theta^{\prime}}}\left( 0 \right) \hfill \\ \end{aligned}$$

where $Re_{x} = \frac{ux}{{\nu_{l} }}$ is local Reynolds number.”

now reads:

“The expressions of dimensionless local friction factors ($Sf_{x} \& Sf_{y}$), local Nusselt number $\left( {Nu_{x} } \right)$ and local Sherwood number $\left( {Sh_{x} } \right)$ are

19 $$\begin{gathered} Re_{x}^{0.5} Sf_{x} = {\Psi }_{2} f^{{{\prime \prime }}} \left( 0 \right), \hfill \\ Re_{x}^{0.5} Sf_{y} = {\Psi }_{2} g^{{\prime }} \left( 0 \right), \hfill \\ Re_{x}^{ - 0.5} Nu_{x} = - \frac{{{\Psi }_{4} \left( {1 + Rd} \right)}}{\theta \left( 0 \right)}, \hfill \\ Re_{x}^{ - 0.5} Sh_{x} = - {\Theta }^{{\prime }} \left( 0 \right) \hfill \\ \end{gathered}$$

where $Re_{x} = \frac{{xu_{w} }}{{\nu_{l} }}$ is local Reynolds number.”

24 $$y_{5}^{{\prime }} = - \frac{{{\Psi }_{2} }}{{{\Psi }_{1} }}\left( {2Roy_{2} + y_{2} y_{4} - y_{1} y_{5} } \right),$$

now reads:

24 $$y_{5}^{{\prime }} = \frac{{{\Psi }_{2} }}{{{\Psi }_{1} }}\left( {2Roy_{2} + y_{2} y_{4} - y_{1} y_{5} } \right),$$

26 $$y_{7}^{{\prime }} = \frac{{ - \Pr \left\{ {{\Psi }_{3} y_{1} y_{7} + Nby_{7} y_{9} + Nt\left( {y_{7} } \right)^{2} + {\Psi }_{2} Ec\left( {x_{3}^{2} + x_{2}^{2} } \right)} \right\}}}{{{\Psi }_{4} + Rd}},$$

now reads:

26 $$y_{7}^{{\prime }} = \frac{{ - \Pr \left\{ {{\Psi }_{3} y_{1} y_{7} + Nby_{7} y_{9} + Nt\left( {y_{7} } \right)^{2} + {\Psi }_{2} Ec\left( {y_{3}^{2} + y_{5}^{2} } \right)} \right\}}}{{{\Psi }_{4} + Rd}},$$

28 $$y_{9}^{{\prime }} = - LePry_{1} y_{10} + \left( {\frac{Nt}{{Nb}}} \right)\left( {\frac{{\Pr \left\{ {{\Psi }_{3} y_{1} y_{7} + Nby_{7} y_{9} + Nt\left( {y_{7} } \right)^{2} + {\Psi }_{2} Ec\left( {x_{3}^{2} + x_{2}^{2} } \right)} \right\}}}{{{\Psi }_{4} + Rd}}} \right),$$

now reads:

28 $$y_{9}^{{\prime }} = - LePry_{1} y_{9} + \left( {\frac{Nt}{{Nb}}} \right)\left( {\frac{{\Pr \left\{ {{\Psi }_{3} y_{1} y_{7} + Nby_{7} y_{9} + Nt\left( {y_{7} } \right)^{2} + {\Psi }_{2} Ec\left( {y_{3}^{2} + y_{5}^{2} } \right)} \right\}}}{{{\Psi }_{4} + Rd}}} \right),$$

Finally, in the Numerical technique section, under the subheading ‘Interpretation of the Outcomes’,

“A diminishing trend of nanoparticle concentration $\left( {{\Theta }\left( \zeta \right)} \right)$ is perceived for advanced values of Nb, while, an opposite trend is observed for temperature $\left( {\theta \left( \zeta \right)} \right)$ profile.”

now reads:

“A diminishing trend of nanoparticle volume fraction (Θ(ζ)) is perceived for advanced values of Nb, while, an opposite trend is observed for temperature (θ(ζ)) profile.”

The original Article has been corrected.

